# Organ Damage and Hepatic Lipid Accumulation in Carp (*Cyprinus carpio* L.) after Feed-Borne Exposure to the Mycotoxin, Deoxynivalenol (DON)

**DOI:** 10.3390/toxins6020756

**Published:** 2014-02-21

**Authors:** Constanze Pietsch, Carsten Schulz, Pere Rovira, Werner Kloas, Patricia Burkhardt-Holm

**Affiliations:** 1 Man-Society-Environment, Department of Environmental Sciences, University of Basel, Vesalgasse 1, Basel CH-4051, Switzerland; E-Mail: patricia.holm@unibas.ch; 2 GMA Society/Association for Marine Aquaculture Ltd., Hafentörn 3, Büsum D-25761, Germany; E-Mail: cschulz@tierzucht.uni-kiel.de; 3 Christian Albrechts-University of Kiel, Institute for Animal Breeding and Husbandry, Olshausestr. 40, Kiel 24098, Germany; 4 Forest Sciences Centre of Catalonia (CTFC), Pujada del Seminari s/n, Solsona E-25280, Spain; E-Mail: pere.rovira@ctfc.es; 5 Department of Ecophysiology and Aquaculture, Leibniz-Institute of Freshwater Ecology and Inland Fisheries, Mueggelseedamm 310, Berlin D-12587, Germany; E-Mail: werner.kloas@igb-berlin.de; 6 Department of Biological Sciences, University of Alberta, CW 405 Biological Sciences Building, Edmonton, AB T6G 2E9, Canada

**Keywords:** liver damage, oxidative stress, nutrient allocation, aquaculture

## Abstract

Deoxynivalenol (DON) frequently contaminates animal feed, including fish feed used in aquaculture. This study intends to further investigate the effects of DON on carp (*Cyprinus carpio* L.) at concentrations representative for commercial fish feeds. Experimental feeding with 352, 619 or 953 μg DON kg^−1^ feed resulted in unaltered growth performance of fish during six weeks of experimentation, but increased lipid peroxidation was observed in liver, head kidney and spleen after feeding of fish with the highest DON concentration. These effects of DON were mostly reversible by two weeks of feeding the uncontaminated control diet. Histopathological scoring revealed increased liver damage in DON-treated fish, which persisted even after the recovery phase. At the highest DON concentration, significantly more fat, and consequently, increased energy content, was found in whole fish body homogenates. This suggests that DON affects nutrient metabolism in carp. Changes of lactate dehydrogenase (LDH) activity in kidneys and muscle and high lactate levels in serum indicate an effect of DON on anaerobic metabolism. Serum albumin was reduced by feeding the medium and a high dosage of DON, probably due to the ribotoxic action of DON. Thus, the present study provides evidence of the effects of DON on liver function and metabolism.

## 1. Introduction

Deoxynivalenol (DON) is a trichothecene mycotoxin that is commonly known to be produced by *Fusarium* fungi, but also fungal species, such as *Myrothecium*, *Cephalosporium*, *Verticimonosporium* and *Stachybotrys* [1]. The toxic effects of DON in mammals include diarrhoea, emesis and malabsorption of nutrients [2,3,4]. A recommended guidance value for DON in compound feed stuff of 5 mg kg^−1^ DON was established by the European Commission (2006/576/EC) [5]. As far as it is known, fish feeds do not exceed this level, although recent research has shown that DON frequently can be observed in fish feeds at concentrations of up to 825 μg kg^−1^ [6]. DON affects fish growth performance and health. For example, exposure to DON resulted in the reduction of growth performance in salmonids [7,8]. These investigations on salmonids also showed that histopathological changes and lesions in the liver of fish, including the altered appearance of hepatocytes, subcapsular edema and fat accumulation, occurred upon feeding with DON-contaminated diets at concentrations of 1.4 mg kg^−1^ and higher [8]. Lipid accumulation in liver tissue is a common problem in aquaculture and can be caused by parasites [9], inadequate nutrition [10,11,12], pesticides [13,14,15] and toxins [16]. Evidence for changes of nutritional status in fish has only been shown for rainbow trout treated with 2.6 mg kg^−1^ DON for 56 days [8], whereby crude protein values were reduced by DON compared to control fish. The effects of DON on the immune system of fish have only been shown for carp at even lower DON concentrations of 352 to 953 μg DON kg^−1^ feed [17].

In the last few years, many studies have been focused on fish health interactions with dietary management. In aquaculture, fish nutrition is critical, and the applied feed contributes up to more than 60% of the total production costs [18]. Thus, nutritional and economical optimization of dietary compositions for certain fish species supports the expanding aquaculture sector. The utilization of plant products in aquaculture feed has been increasing in the last few years tremendously, as the supply of conventional feed sources, e.g., fish meal, is limited, and prices are consequently rising. With increasing plant material utilization in aquaculture, the risk for feed borne exposure to DON increases. Therefore, further understanding of dietary DON on the health and nutritional value of carp (*Cyprinus carpio*), as an aquaculture species of high global relevance, is needed to guarantee fish health and a safe product for human nutrition.

The aim of the present study was therefore the evaluation of various DON feed dosages on the growth performance and health status of juvenile carp. The present study is a part of a feeding study in which stress and immune responses already have been evaluated and published elsewhere [17]. The analyses reported in the present study were conducted on preserved samples from this trial, to further elaborate the detrimental health effects of DON observed in the experiment. The main findings reported in the previous study were that fish fed the diet containing DON showed reduced immune parameters. The present study intended to investigate the effects of DON on liver condition and metabolism more closely.

## 2. Results and Discussion

### 2.1. Composition of the Diet and Growth Performance

The experimental diets were prepared as shown in Table 1. The inclusion of ingredients has been chosen to meet the nutritional requirements of carp [19]. The experimental diets differed only in their DON content.

**Table 1:** The ingredients (percent of inclusion) used for preparations of experimental fish diets. DON, deoxynivalenol.

Ingredient	Basal Feed	Low DON	Medium DON	High DON
Fish meal	30.0	30.0	30.0	30.0
Blood meal	12.5	12.5	12.5	12.5
Casein	12.0	12.0	12.0	12.0
Dextrose	13.0	13.0	13.0	13.0
Potato starch	21.1	21.1	21.1	21.1
Fish oil	10.4	10.4	10.4	10.4
Vitamins^1^	0.5	0.5	0.5	0.5
Minerals^1^	0.5	0.5	0.5	0.5
DON (mg kg^−1^)	0	0.352	0.619	0.953

Notes: Vitamin and mineral mix (Spezialfutter Neuruppin-VM BM 55/13 no. 7318): vitamin A, 12,000 I.E.; vitamin D3, 1,600 I.E; vitamin E, 160 mg; vitamin K3, 6.4 mg; vitamin B1, 12 mg; vitamin B2, 16 mg; vitamin B6, 12 mg; vitamin B12, 26.4 μg; nicotinic acid, 120 mg; biotin, 800 μg; folic acid, 4.8 mg; pantothenic acid, 40 mg; inositol, 240 mg; vitamin C, 160 mg; antioxidants (BHT), 120 mg; iron, 100 mg; zinc, 24 mg; manganese, 16 mg; cobalt 0.8 mg; iodine, 1.6 mg; selenium, 0.08 mg.

The nutritional compositions of experimental diets are given in Table 2, showing that no nutritional differences between the diets occurred. The diets were formulated to be isonitrogenous (44.58%–45.91% crude protein) and isocaloric (22.26–22.51 MJ kg^−1^ dry matter).

After four weeks of feeding, two tanks containing six fish each were sampled, whereas two similar treated tanks containing 12 fish were fed the uncontaminated diet for a further two weeks and sampled thereafter. The growth performance, serum parameters and biochemical composition of all fish were analysed.

**Table 2:** The composition of the experimental fish feeds. The values are given as the means ± SD of two independent determinations of the same feed batch. NFE, nitrogen-free extract.

Composition	Basal Feed	Low DON	Medium DON	High DON
Dry matter (% wet matter)	91.70 ± 0.01	91.58 ± 0.02	91.66 ± 0.05	91.68 ± 0.04
Crude protein (% dry matter)	44.58 ± 0.28	45.91 ± 0.17	45.14 ± 0.06	44.89 ± 0.48
Crude lipid (% dry matter)	14.61 ± 0.03	14.56 ± 0.07	14.63 ± 0.01	14.96 ± 0.20
NFE (% dry matter)	33.15 ± 0.36	32.13 ± 0.18	32.60 ± 0.01	32.47 ± 0.34
Crude ash (% dry matter)	7.66 ± 0.01	7.40 ± 0.03	7.64 ± 0.05	7.68 ± 0.05
Gross energy (MJ kg^−1^ dry matter)	22.51 ± 0.07	22.46 ± 0.01	22.26 ± 0.02	22.42 ± 0.01

Feeding carp the experimental diets at a restricted daily basis of 2% of body weight resulted in increased fish body weights after four weeks (Table 3). Compared to the initial weight of fish, significant weight gain was observed for the groups fed the DON-contaminated diets, but not for the control group. However, individual specific growth rates (SGR), calculated as shown in Equation (1) were not found to be different between treatment groups after four weeks of feeding (mean ± SEM: control fish: 1.31 ± 0.11; low dose: 1.13 ± 0.20; medium dose: 1.71 ± 0.31; high dose: 1.50 ± 0.06, respectively).

SGR = (ln final weight − ln initial weight)/days of experiments × 100 (1)

**Table 3:** The growth performance of experimental fish after four weeks of DON feeding; *n* = 12 each.

Growth Parameters	Basal Feed	Low DON	Medium DON	High DON
Initial Weight (g)	37.23 ± 6.31	36.36 ± 6.67	36.23 ± 8.00	34.46 ± 4.05
Final Weight (g)	48.38 ± 7.48	51.86 ± 5.50	52.71 ± 6.37	56.40 ± 10.63
Final Total Length (cm)	14.11 ± 0.61	14.64 ± 0.52	14.76 ± 0.66	14.62 ± 0.99
Final Condition Factor	0.016 ± 0.000	0.016 ± 0.000	0.016 ± 0.000	0.016 ± 0.000

Furthermore, condition factors were not different between treatment groups after four weeks of feeding and also after recovery for additional weeks (Table 3 and Table 4). The final weight of fish after the recovery phase was also not significantly influenced by DON application (Table 4). Individual specific growth rates were also not different between treatment groups after the recovery phase (mean ± SEM: control fish: 1.26 ± 0.26; low dose: 1.62 ± 0.23; medium dose: 1.19 ± 0.30; high dose: 1.26 ± 0.25, respectively). However, the comparison of final weights to the initial weights at the start of the experiments showed a significant difference for the control group and the fish fed the low dose diet, but not for the fish fed the higher DON doses.

**Table 4:** The growth performance of experimental fish after four weeks of DON feeding with an additional two weeks of recovery; *n* = 12 each.

Growth Parameters	Basal Feed	Low DON	Medium DON	High DON
Initial Weight (g)	38.24 ± 5.51	33.46 ± 4.42	36.71 ± 5.01	38.18 ± 8.77
Final Weight (g)	65.58 ± 10.42	57.56 ± 8.36	50.99 ± 9.15	53.72 ± 9.83
Final Total Length (cm)	14.11 ± 0.61	14.64 ± 0.52	14.76 ± 0.66	14.62 ± 0.99
Final Condition Factor	0.015 ± 0.000	0.015 ± 0.000	0.015 ± 0.000	0.014 ± 0.000

Reduced intake of DON-contaminated feed leading to reduced weight gain was reported in mice [20], but not in the present study. Thus, it is unlikely that feed deprivation was a factor in the effects on carp metabolism, as fish were observed to ingest the entire feed ration. Nevertheless, Atlantic salmon showed reduced weight gain after 15 weeks of feeding of 3.7 mg DON per kilogram of feed, while rainbow trout revealed similar responses to 0.3 to 2.6 mg DON per kilogram of feed after 56 days of feeding [7,8]. In contrast, a previous study on zebrafish showed that treatment of fish with feed-borne DON concentrations of up to 3 mg per kilogram of feed did not result in effects on weight gain [21]. Thus, our results together with the study of Sanden *et al.* [21] suggest that weight gain is not a sensitive parameter when the effects of DON are investigated in cyprinids.

### 2.2. Histology

Prussian blue staining in liver tissue did not result in extensive staining of macrophages in liver tissues in the treated groups and in the control group. Therefore, results are not reported here. PAS reaction was positive in the liver of all fish. However, PAS staining did not indicate significant differences between control fish and DON-treated fish with respect to chrominance, RGB_max_ and luminosity values (Table 5).

**Table 5:** The histological estimation of glycogen in PAS-stained liver sections of experimental fish after four weeks of DON feeding; *n* = 6 each, calculated from five pictures from each slide.

Color Properties of Sections	Basal Feed	Low DON	Medium DON	High DON
Chrominance	50.1 ± 2.8	50.5 ± 1.2	46.7 ± 3.4	47.4 ± 3.0
RGB_max_	135.1 ± 1.5	133.8 ± 2.4	129.9 ± 4.1	131.4 ± 2.4
Luminosity	23.7 ± 1.5	24.1 ± 0.9	23.1 ± 2.0	23.2 ± 1.7

**Figure 1:**
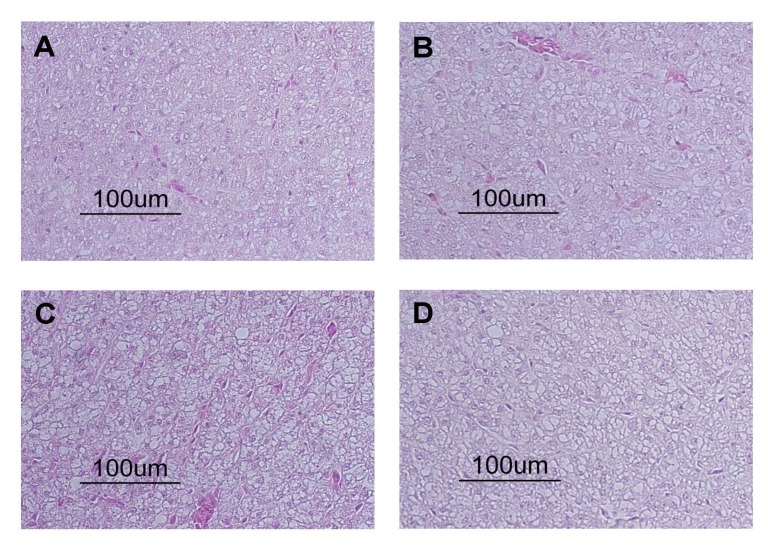
Histological sections of liver tissue from control fish (**A**) and DON-treated fish [low dose (**B**), medium dose (**C**) and high dose (**D**)].

However, the examination of haematoxylin and eosin (HE)-stained sections revealed significant differences between the condition of the liver tissue of control fish and DON-treated fish (Table 6). DON-treated fish showed significantly increased fat disposition (Figure 1) and severe hyperaemia, whereas no significant difference was found when tissue lesions and the degree of vacuolization were recorded. The observation of the dilatation of sinusoids revealed a significant difference between control fish and fish treated with the low and medium DON diet. In rainbow trout fed 1.4 mg kg^−1^ DON for 15 weeks, congestion in liver tissue was observed [8].

In addition, feeding 2.6 mg kg^−1^ DON in the same study led to fatty infiltration in liver. Histological changes, including fat deposition, have also been found in DON-treated carp in the present study. Liver damage was probably caused by the occurrence of oxidative stress (as indicated by the lipid peroxidation assay) together with apoptotic loss of cell integrity and disturbance of nutrient metabolism. Liver damage also often leads to the occurrence of increased liver fat content, as is reported for fish recovering from being fed the high-dose DON feed for four weeks (Table 6).

**Table 6:** The histological condition of haematoxylin and eosin (HE)-stained liver tissue after DON feeding and a two-week recovery phase (mean ± standard errors of six fish per treatment group; each fish was analysed by using 10 fields taken from two slides (0 = no alterations, 1 = mild alterations, 2 = moderate alterations, 3 = severe alterations); means with the same letter (^a^ and/or ^b^) are not significantly different from each other [significance tested with Mann-Whitney U-tests, *p* < 0.05)].

Histological Alteration	Basal Feed	Low DON	Medium DON	High DON
DON-treated:				
Lesions	0.03 ± 0.03	0.20 ± 0.13	0.14 ± 0.05	0.35 ± 0.15
Fat aggregation	0.92 ± 0.07 ^a^	1.57 ± 0.28 ^a,b^	1.94 ± 0.25 ^b^	1.80 ± 0.19 ^b^
Hyperaemia	1.00 ± 0.12 ^a^	2.02 ± 0.26 ^b^	1.90 ± 0.26 ^b^	1.90 ± 0.23 ^b^
Vacuolization	1.17 ± 0.06	1.77 ± 0.32	1.84 ± 0.32	1.85 ± 0.20
Dilation of sinusoids	1.20 ± 0.16 ^a^	1.87 ± 0.07 ^b^	1.82 ± 0.13 ^b^	1.60 ± 0.16 ^a,b^
Recovery:				
Lesions	0.02 ± 0.02	0.28 ± 0.15	0.12 ± 0.07	0.18 ± 0.09
Fat aggregation	0.50 ± 0.18 ^a^	1.85 ± 0.26 ^b^	1.47 ± 0.31 ^a,b^	1.63 ± 0.29 ^b^
Hyperaemia	1.26 ± 0.11 ^a^	1.70 ± 0.17 ^a,b^	1.37 ± 0.19 ^a,b^	2.08 ± 0.23 ^b^
Vacuolization	0.54 ± 0.22	1.45 ± 0.41	1.42 ± 0.38	1.48 ± 0.38
Dilation of sinusoids	1.44 ± 0.13 ^a^	2.10 ± 0.12 ^b^	1.78 ± 0.18 ^a,b^	1.92 ± 0.11 ^a,b^

### 2.3. Lipid Peroxidation

**Figure 2:**
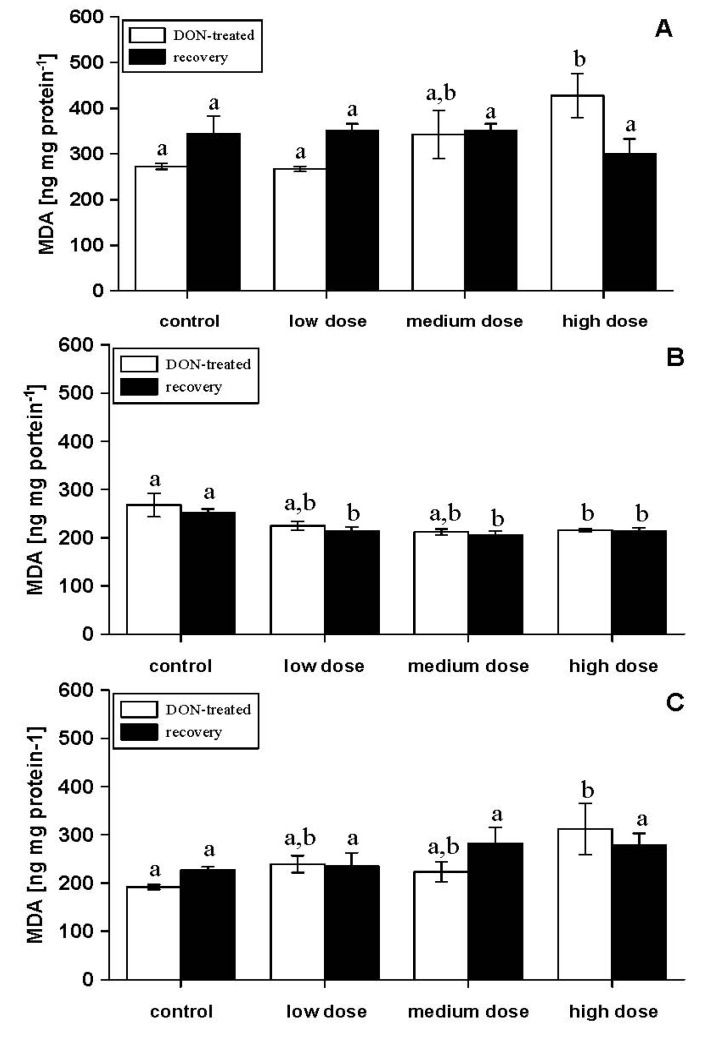
Lipid peroxidation measured as malondialdehyde (MDA; ng mg protein^−1^) in different tissues [(**A**) head kidney, (**B**) trunk kidney, (**C**) spleen] in experimental fish with four weeks of DON feeding (DON-treated) and DON-fed fish with an additional two weeks of recovery (recovery); mean ± SEM; means with the same letter (^a^ and/or ^b^) are not significantly different from each other (significance tested with Mann-Whitney U-tests, *p* < 0.05).

Measurement of lipid peroxidation indicated increased membrane damages in fish fed the diet with the high-dose DON compared to control fish (Figure 2A). This was no longer observed after the recovery phase of two weeks. A similar pattern was observed in spleen (Figure 2C). In contrast, lipid peroxidation in trunk kidney was reduced in the group treated with the high dose DON feed after four weeks of feeding and in all DON-treated fish after the recovery (Figure 2B).

**Figure 3:**
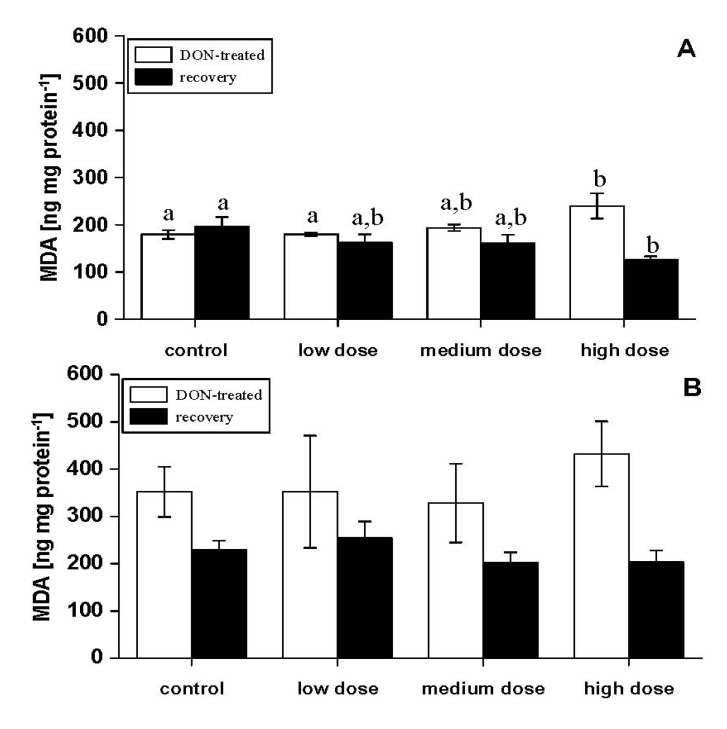
Lipid peroxidation measured as malondialdehyde (MDA; ng mg protein^−1^) in different tissues [(**A**) liver, (**B**) white muscle] in experimental fish with four weeks of DON feeding (DON-treated) and DON-fed fish with an additional two weeks of recovery (recovery); mean ± SEM; means with the same letter (^a^ and/or ^b^) are not significantly different from each other (significance tested with Mann–Whitney U-tests, *p* < 0.05).

Lipid peroxidation in liver was enhanced in fish treated with the high-dose feed after four experimental weeks and significantly reduced after the recovery phase compared to control fish (Figure 3A). Lipid peroxidation in muscle samples showed no differences, due to DON treatment (Figure 3B). Oxidative stress in these carp has already been indicated by the elevation of antioxidative enzymes in erythrocytes [17] and probably also contributed to the damage to several organs of DON-treated fish. Oxidative stress and lipid peroxidation due to the *Fusarium* toxins, deoxynivalenol and zearalenone, have frequently been shown in mammalian cell cultures and farm animals [22,23,24].

### 2.4. Measurement of LDH Activity in Different Tissues

The activity of the lactate dehydrogenase (LDH) showed differences in the kidneys after four weeks of DON feeding and also after additional two weeks of recovery but not in liver and spleen (Table 7). During DON feeding LDH activity was increased in the kidneys of all DON-fed fish compared to control fish which indicates increased anaerobic metabolism. However, a lower LDH activity in the recovery phase was observed between fish fed the highest DON diet and the control fish. The significant difference in LDH activity in muscle after four weeks of feeding the highest dose of DON was no longer observable after the recovery of two weeks. The observation that lactate levels are not increased in muscle samples although LDH activities are decreased in fish treated with the high dose DON diet, supports the hypothesis that lactate is transferred from white muscle via the blood stream before it accumulates.

### 2.5. Biochemical Body and Organ Composition

Investigation on whole body composition revealed that the fat content in whole body homogenates of fish treated with the medium- and high-dose DON diet was increased compared to control fish (Table 8), whereas in fish treated with the low dose DON diet, only a tendency for increased fat content was found (*p* = 0.065). Still, a significant relationship between the toxin concentrations in the experimental feeds and lipid content was found (Spearman correlation coefficient, 0.498, and significance, *p* = 0.013).

**Table 7:** Lactate dehydrogenase (LDH; mU mg protein^−1^) activity in tissue homogenates after DON feeding and a two-week recovery phase; *n* = 6; mean ± SEM; means with the same letter (^a^ and/or ^b^) are not significantly different from each other (significance tested with Mann-Whitney U-tests, *p* < 0.05).

LDH Activity	Basal Feed	Low DON	Medium DON	High DON
DON-treated:				
head kidney	31.58 ± 4.77 ^a^	71.55 ± 3.63 ^b^	61.22 ± 5.11 ^b^	51.89 ± 6.30 ^b^
trunk kidney	34.34 ± 5.18 ^a^	77.79 ± 3.95 ^b^	66.23 ± 4.55 ^b,c^	53.16 ± 6.84 ^a,c^
spleen	2.64 ± 0.25	2.97 ± 0.47	2.90 ± 0.34	2.92 ± 0.43
liver	275.23 ± 61.86	370.96 ± 66.37	176.58 ± 40.47	264.67 ± 56.73
white muscle	38.28 ± 13.68 ^a^	17.51 ± 5.77 ^a,b^	20.86 ± 7.39 ^a,b^	3.12 ± 1.81 ^b^
Recovery:				
head kidney	51.21 ± 4.97 ^a^	52.54 ± 6.65 ^a^	57.12 ± 9.96 ^a^	13.34 ± 2.77 ^b^
trunk kidney	54.58 ± 6.08 ^a^	65.33 ± 6.10 ^a^	60.70 ± 13.15^a^	19.89 ± 4.50 ^b^
spleen	10.88 ± 0.99	9.74 ± 0.90	7.92 ± 0.59	12.96 ± 2.86
liver	496.02 ± 118.64	669.79 ± 113.86	510.05 ± 106.11	344.31 ± 89.97
white muscle	17.73 ± 4.85	12.35 ± 4.57	22.29 ± 3.90	25.91 ± 3.92

After two additional weeks of recovery, all DON-treated fish showed lower lipid levels than the control fish (Table 9). These results were paralleled by significant differences in the energy content of the whole body homogenates. After recovery, the ash contents of the whole body homogenates were also found to be different in fish fed the high-dose DON diet compared to control fish (Table 9).

In the recovered fish, the ash content correlated with the lipid content negatively (Spearman correlation coefficient, −0.568; significance, *p* = 0.002). Moreover, a significant relationship between the toxin concentrations in the experimental feeds and the lipid and ash content was found (Spearman correlation coefficient, −0.792 and 0.568, and significance, *p* < 0.000 and *p* = 0.007, respectively) in fish after the recovery phase. This led to the investigation of similar parameters in samples of liver tissue and white dorsal musculature to allow a possible explanation for these observations. 

Impairment of the intestinal nutrient uptake by DON was reported for mammalian systems [25,26,27]. Since the liver is an important metabolic organ that processes nutrients from feed, one would expect effects on the nutritional status of liver tissue if such impairment of intestinal nutrient uptake would also be present in the experimental carp.

Therefore, the biochemical composition of liver tissue of carp was analysed. Dry matter values of liver tissue were comparable to the values reported for other cyprinid species, such as freshwater major carp, *Catla catla* [28]. No significant differences in the dry matter of DON-treated fish compared to control fish were observed after four weeks of experiments, but an additional two weeks of recovery led to significantly different dry matter incorporations between fish fed the low-dose DON feed and the control fish (Table 10). Liver total lipid contents are higher than in the whole body homogenates. This seems feasible, since liver in carp is known to be important for the storage of lipids. Moreover, in fish treated with the highest concentration of DON, the lipid content in liver tissue was significantly increased after the recovery phase compared to control fish. This is in contrast to the whole body lipid levels of DON-treated fish after the recovery phase and indicates that liver damage was accompanied by hepatic lipid accumulation, which also influenced the lipid balance of the entire body.

**Table 8:** The whole body composition of experimental fish two weeks after DON feeding (recovery phase); *n* = 6; mean ± SEM; means with the same letter (^a^ and/or ^b^) are not significantly different from each other (significance tested with Mann-Whitney U-tests, *p* < 0.05).

Whole Body Composition	Basal Feed	Low DON	Medium DON	High DON
Crude lipid content (% dry matter)	24.83 ± 1.85 ^a^	29.33 ± 1.36 ^a,b^	32.17 ± 0.95 ^b^	30.33 ± 0.95 ^b^
Crude ash (% dry matter)	8.56 ± 1.22	8.72 ± 0.19	8.03 ± 1.01	9.37 ± 0.34
Energy content (MJ kg^−1^ dry matter)	24.32 ± 0.47 ^a^	25.27 ± 0.34 ^a,b^	25.78 ± 0.25 ^b^	25.25 ± 0.31 ^a,b^

**Table 9:** The whole body composition of experimental fish two weeks after DON feeding (recovery phase); *n* = 6; mean ± SEM; means with the same letter (^a^ and/or ^b^) are not significantly different from each other (significance tested with Mann-Whitney U-tests, *p* < 0.05).

Whole Body Composition	Basal Feed	Low DON	Medium DON	High DON
Crude lipid content (% dry matter)	33.17 ± 0.75 ^a^	30.25 ± 0.48 ^b^	29.60 ± 1.08 ^b^	25.00 ± 2.71 ^b^
Crude ash (% dry matter)	8.23 ± 0.35 ^a^	8.46 ± 0.34 ^a,b^	9.37 ± 0.34 ^a,b^	9.98 ± 0.53 ^b^
Energy content (MJ kg^−1^ dry matter)	25.63 ± 0.12 ^a^	25.40 ± 0.19 ^a,b^	25.02 ± 0.28 ^a,b^	24.64 ± 0.23 ^b^

**Table 10:** The composition of liver samples after four weeks of DON feeding and after the recovery phase of two weeks; *n* = 6; mean ± SEM; means with the same letter (^a^ and/or ^b^) are not significantly different from each other (significance tested with Mann-Whitney U-tests, *p* < 0.05).

Liver Composition	Basal Feed	Low DON	Medium DON	High DON
DON-treated:				
Dry matter (% wet matter)	25.9 ± 0.8	22.9 ± 2.2	25.6 ± 0.8	27.0 ± 1.4
Total lipids (% wet matter)	15.0 ± 1.3	14.5 ± 1.2	16.2 ± 1.7	17.3 ± 1.7
Free glucose (mM g^−1^ wet matter)	1.16 ± 0.27	0.87 ± 0.14	1.19 ± 0.08	0.91 ± 0.19
Lactate (mM g^−1^ wet matter)	1.75 ± 0.48	3.05 ± 1.29	3.77 ± 2.03	3.56 ± 1.87
AST (U mg protein^−1^)	90.3 ± 18.2	60.2 ± 20.5	81.7 ± 8.2	89.1 ± 16.6
ALT (U mg protein^−1^)	1.43 ± 0.14 ^a^	2.61 ± 0.98 ^a,b^	2.95 ± 0.51 ^b^	2.73 ± 0.49 ^a,b^
Ascorbate (μM g^−1^ wet matter)	81.7 ± 15.7	63.5 ± 8.3	71.5 ± 14.8	54.3 ± 8.8
Recovery:				
Dry matter (% wet matter)	25.1 ± 0.5 ^a^	28.9 ± 1.4 ^a^	27.5 ± 1.8 ^a,b^	25.8 ± 1.7 ^a,b^
Total lipids (% wet matter)	17.7 ± 1.2 ^a^	19.2 ± 1.3 ^a,b^	18.7 ± 0.8 ^a,b^	23.6 ± 4.1 ^b^
Free glucose (mM g^−1^ wet matter)	0.73 ± 0.10	0.96 ± 0.17	0.91 ± 0.12	0.99 ± 0.21
Lactate (mM g^−1^ wet matter)	1.36 ± 0.35 ^a^	3.74 ± 1.13 ^a,b^	6.48 ± 2.02 ^b^	2.85 ± 0.80 ^a,b^
AST (U mg protein^−1^)	98.5 ± 22.1	96.5± 27.5	81.7 ± 8.2	99.7 ± 10.8
ALT (U mg protein^−1^)	2.47 ± 0.23	2.88 ± 0.22	2.79 ± 0.11	3.03 ± 0.26
Ascorbate (μM g^−1^ wet matter)	51.8 ± 6.5	58.8 ± 7.0	64.9 ± 7.5	62.2 ± 7.6

Liver glucose levels were not significantly different between treatment groups. It was suggested that amino acids are a superior energy sources to glucose for carp [29]. It has also been shown for carps under starvation that the conversion of lipid to glycogen in liver tissue was accompanied by an increase in blood glucose levels [30]. No changes of blood glucose (Table 11) and lipid (Table 10) in liver tissue were observed in DON-treated fish, which suggests that blood homeostasis was not subjected to a fasting-like status, due to an impairment of nutrient uptake in the intestine.

Liver glucose, lactate and ascorbate concentrations were not influenced by DON feeding and did not correlate with toxin concentrations in the experimental feeds. Although ascorbic acid has been shown to prevent the hemolytic action of DON on rat erythrocytes to some extent [22], ascorbate levels in carp remained unchanged, which indicated that ascorbate alone is not sufficient to prevent damage due to DON exposure. Liver alanine aminotransferase (ALT) activity was increased by treatment with the medium-dose DON diet for four weeks compared to control fish, while the other DON-treated groups showed no significant difference of this enzyme activity compared to the control group, probably due to higher individual variation (coefficient of variance (*CV*) for control fish of 24.6 *versus* *CV*s of 92.2, 42.3 and 51.0 for fish treated with the low-, medium- and high-dose, respectively, while the *CV*s for all fish in the recovery phase ranged from 9.5 to 23.2). ALT activity also correlated with the mycotoxin concentrations in feed (Spearman correlation coefficient, −0.409; significance, *p* = 0.047). This indicates damage to liver tissue, as has already been observed in carp under chemical stress [31]. In contrast, aspartate aminotransferase (AST) activity was not significantly influenced by DON feeding, which indicates that the rate of amino acid transformations via transamination is not influenced.

A considerable amount of the fish consists of white musculature, which shows low levels of myoglobin and is mostly used for burst swimming [32]. Swimming performance is known to lead to the utilization of nutrients from blood circulation and white and red musculature [33]. Several factors further influence the chemical body composition. For example, the genetic background of carp determined the dry matter content of the fillet, as has been shown for different crossbreds of common carp, accounting for 19% to 28% [34,35,36]. With respect to common mirror carps, a value for dry matter of 22.0% was observed [36]. The dry matter of the carp used in our study corresponds to this value and was only influenced by feeding the low-dose DON diet (Table 12). 

As expected, the lipid content in carp muscle was found to be low in the present study, and it was not influenced by DON feeding. Different crossbreds of common carp showed lipid contents in the fillet of up to 9.9%, with mirror carps showing 2.4% lipid in fillet [36]. Low muscle lipid contents ranging from 0.5% to 2.6% have also been noted by another study [34]. However, slightly higher values, ranging from 2.9% to 5.2%, have been reported elsewhere [35,37]. Differences in the chemical composition of carp musculature can be due to the influence of rearing conditions [38,39], the influence of age [35,36,39] and differences in the composition of the diet [37,39,40,41], and these factors should be considered when the values for the present study are compared to other studies. For example, in the study of Steffens and Wirth [40], the addition of 10% different lipid sources in the diet led to 2.2 to 2.5% lipid in dorsal muscle of carp, which corresponds to the values in the present study.

The significant difference in the dry matter of white muscle of fish fed the low-dose diet compared to the control fish cannot be explained at the moment. Reductions of dry matter in the musculature can be caused by the depletion of tissue nutrient contents, which are compensated for by increasing the water content [29]. However, even after calculation of the dry weight lipid contents in white muscle, the samples do not show a significant influence of DON. Thus, the reason for this observation remains obscure.

Muscle glucose shows no differences of the DON treatment of fish. The glucose concentration in red musculature has been reported to be rather independent of blood glucose levels [42], but the aerobic glucose utilization was still assumed to be relying by approximately 30% on glucose in circulation. In contrast, white musculature in fish is known to largely depend on anaerobic glycogenolysis for energy liberation [43]. Lactate in carp white musculature ranged from four to 9 mM [44], even after exhaustive exercise. The lactate levels in muscle tissue in the present study were a bit lower and were not influenced by DON feeding. This might be due to the fact that even after severe hypoxic stress, lactate did not accumulate in white muscle, but was probably transferred out of the tissue [45].

Ascorbate levels in white muscle samples strongly depend on the supply via the diet and have been reported to be low in fish musculature [46]. This corresponds to the present study, although higher values have been reported for carp previously [47]. This study also reported an influence of the mycotoxin, sterigmatocystin, on ascorbate levels in the white musculature of carp, which was not observed after DON feeding of carp in the present study. Similar to the ascorbate levels in liver tissue, the lack of influence of DON on their concentration in muscle further indicates that ascorbate as an endogenous antioxidant does not prevent the detrimental effects of DON on carp.

### 2.6. Serum Parameter

Our study demonstrates a large variation of biochemical serum parameters, which are known to be affected by many endogenous and exogenous factors, such as age, health condition, nutrition or stress, including chemical stress [48,49].

Glucose levels in the experimental fish in the present study were comparable to values for unstressed carp reported previously [48,49]. Glucose concentrations in serum remained unchanged in fish treated with DON for four weeks (Table 11). A direct influence of DON on glucose metabolism has been shown in the human epithelial intestinal cell line, HT-29-D4, and in jejunum of chicken; however, cholesterol metabolism remained unchanged in this cell line [26,27]. No evidence for a similar impairment of glucose from the diet could be observed in the present study, although a possible decrease in blood glucose might also have been compensated for in DON-treated fish. Similar to other fish, carp are known to maintain blood glucose levels, even after prolonged starvation [29].

Serum lactate levels in control fish are comparable to values in unstressed fish in other studies [49,50]. However, lactate values in DON-treated carp were considerably higher. This was probably caused by an activation of gluconeogenesis to maintain levels of circulation glucose. Consequently, elevated serum glucose levels can be caused [49], which has also been observed in fish recovering for the treatment with the medium- and high-dose diet compared to the control fish (Table 11). Thus, it can be assumed that DON affects anaerobic metabolism in carp muscle.

**Table 11:** The serum parameter in DON-treated fish and in fish after a recovery of two weeks; *n* = 6 per group; mean ± SEM; means with the same letter (^a^ and/or ^b^) are not significantly different from each other (significance tested with Mann-Whitney U-tests, *p* < 0.05).

Serum Parameters	Basal Feed	Low DON	Medium DON	High DON
DON-treated:				
Free glucose (μM mL^−1^)	2.22 ± 0.22	2.37 ± 0.19	2.12 ± 0.09	2.53 ± 0.27
Lactate (μM mL^−1^)	8.04 ± 2.87 ^a^	21.85 ± 5.78 ^a,b^	20.50 ± 6.70 ^a,b^	29.96 ± 8.44 ^b^
LDH (mU mg protein^−1^)	15.7 ± 1.0 ^a^	19.8 ± 1.8 ^a,b^	18.6 ± 2.5 ^a,b^	23.6 ± 2.1 ^b^
SDH (mU mg protein^−1^)	11.2 ± 4.4 ^a,b^	30.3 ± 4.8 ^a^	23.1 ± 8.2 ^a,b^	8.5 ± 2.1 ^b^
AST (U mg protein^−1^)	7.1 ± 0.9 ^a^	5.1 ± 0.2 ^a,b^	4.8 ± 0.7 ^b^	10.6 ± 3.8 ^a,c^
ALT (U mg protein^−1^)	0.7 ± 0.2 ^a^	0.7 ± 0.3 ^a^	1.2 ± 0.6 ^a^	0.2 ± 0.0 ^b^
Total protein (mg mL^−1^)	23.05 ± 0.73	23.32 ± 0.64	26.22 ± 1.26	23.41 ± 0.78
Albumin (mg mL^−1^)	19.85 ± 1.49 ^a^	15.14 ± 2.03 ^a,b^	5.42 ± 2.40 ^b^	6.92 ± 3.66 ^b^
Recovery:				
Free glucose (μM mL^−1^)	1.66 ± 0.12 ^a^	2.78 ± 0.28 ^b^	2.84 ± 0.33 ^b^	2.77 ± 0.25 ^b^
Lactate (μM mL^−1^)	4.12 ± 1.08 ^a^	34.87 ± 12.74 ^b^	39.70 ± 8.35 ^b^	19.83 ± 3.70 ^b^
LDH (mU mg protein^−1^)	14.8 ± 1.4 ^a^	18.8 ± 1.0 ^b^	17.4 ± 0.9 ^a,b^	21.9 ± 1.6 ^b^
SDH (mU mg protein^−1^)	32.4 ± 8.8	16.1 ± 3.5	20.3 ± 5.4	34.5 ± 11.7
AST (U mg protein^−1^)	6.4 ± 0.8	5.1 ± 0.2	4.8 ± 0.7	7.9 ± 2.6
ALT (U mg protein^−1^)	0.4 ± 0.1 ^a^	0.6 ± 0.1 ^a^	0.7 ± 0.2 ^a,b^	1.1 ± 0.2 ^b^
Total protein (mg mL^−1^)	21.89 ± 0.73	22.04 ± 0.39	21.47 ± 1.86	29.86 ± 0.57
Albumin (mg mL^−1^)	15.32 ± 1.50	17.22 ± 1.74	16.94 ± 3.75	22.45 ± 2.40

**Table 12:** The composition of samples from dorsal white musculature after four weeks of DON feeding and after the recovery phase of two weeks; *n* = 6; mean ± SEM; means with the same letter (^a^ and/or ^b^) are not significantly different from each other (significance tested with Mann-Whitney U-tests, *p* < 0.05).

Composition of White Muscle	Basal Feed	Low DON	Medium DON	High DON
DON-treated:				
Dry matter (% wet matter)	22.4 ± 2.0 ^a^	19.0 ± 0.7 ^b^	20.9 ± 0.6 ^a,b^	20.5 ± 0.4 ^a,b^
Total lipids (% wet matter)	2.4 ± 0.4	2.2 ± 0.2	2.4 ± 0.1	2.1 ± 0.2
Free glucose (μM g^−1^ wet matter)	2.93 ± 0.39	3.30 ± 0.22	2.95 ± 0.29	2.76 ± 0.29
Lactate (mM g^−1^ wet matter)	3.54 ± 0.37	4.08 ± 0.29	4.56 ± 0.30	3.79 ± 0.21
Ascorbate (μM g^−1^ wet matter)	10.2 ± 0.5	10.0 ± 0.8	10.3 ± 0.5	9.5 ± 0.6
Recovery:				
Dry matter (% wet matter)	19.6 ± 0.9	19.7 ± 0.3	21.2 ± 0.6	19.7 ± 0.5
total lipids (% wet matter)	2.2 ± 0.3	2.5 ± 0.2	2.6 ± 0.2	2.9 ± 0.6
Free glucose (μM g^−1^ wet matter)	3.33 ± 0.34	3.12 ± 0.18	3.89 ± 0.40	3.32 ± 0.48
Lactate (mM g^−1^ wet matter)	3.03 ± 0.11	3.41 ± 0.48	2.95 ± 0.22	2.94 ± 0.31
Ascorbate (μM g^−1^ wet matter)	9.7 ± 0.3	9.3 ± 0.5	9.1 ± 0.7	10.2 ± 0.7

Total protein content in all carp used in the present study was slightly lower in most cases compared to previously reported values in other studies [33,49]. From this, it may be assumed that in general, the nutritional status of the fish was sufficient, since serum total protein levels are known to reflect the nutritional condition of carp [29]. The albumin concentration in the control group was comparable to previously reported values [33]. Although the total protein content of serum remained unchanged by DON feeding, albumin concentrations were significantly reduced in fish fed the medium dose and high dose diets for four weeks. This means that the ratio of albumin to total proteins in control fish of more than 80% is reduced in DON-treated fish to 66%, 21% and 31% in fish treated with the low-dose, medium-dose and high-dose diet, respectively. Which adaptations led to the maintenance of total protein levels, although albumin levels were reduced by DON treatment, remains unknown and should be investigated in future studies. Nevertheless, the effect on serum albumin levels was certainly caused by liver impairment that occurred upon DON treatment. That trichothecenes, including DON, are ribotoxic, targeting the 60S ribosomal subunit, and consequently, impairing protein synthesis and transcription, which leads to apoptosis, has already been reported for leukocytes and other actively proliferating eukaryotic cells of higher vertebrates [51].

In contrast to their activity in liver tissue (Table 10), the activities of AST and ALT in serum were significantly reduced by DON feeding in the medium-dose group and the high-dose group, respectively, compared to control fish (Table 11), and only ALT activity was increased in fish recovering from receiving the high-dose DON diet. This indicates that the rate of amino acid transformation via transamination is slowed down by DON. Lactate dehydrogenase (LDH) activity in serum was found to be increased in fish fed the high-dose feed for four weeks and after two weeks of recovery compared to control fish. Increased LDH in serum of carp, indicating membrane leakage in tissues, has also been shown after exposure to toxic concentrations of pesticides [52]. This parallels the increased lactate levels in DON-treated fish. SDH activity was increased in these fish compared to fish fed the low-dose DON diet. Significant increases of AST, ALT and LDH activities have been observed in carp that have been exposed to handling stress, regular exercise or toxic substances, such as microcystins and cyanide [33,53,54,55,56]. Although correlations between the toxin concentrations in feed and AST activities in serum were not significant in the present study during the feeding period, our results showed a significant correlation of ALT, LDH or SDH with the toxin concentrations in the experimental diets (Spearman correlation coefficient, −0.527, 0.466 and 0.475; significance, *p* = 0.012, 0.022 and 0.025, respectively). Moreover, after the same time period, albumin or lactate concentrations in serum correlated with the toxin concentration in the experimental feeds (Spearman correlation coefficient, −0.678 and 0.453; significance, *p* = 0.000 and 0.034, respectively). Similar results have also been obtained for the recovery phase for the correlation of ALT and LDH with the toxin concentrations previously applied (Spearman correlation coefficient, 0.433 and 0.555; significance, *p* = 0.034 and 0.005, respectively). Furthermore, glucose or lactate concentrations in serum correlated with the toxin concentration in the experimental feeds (Spearman correlation coefficient, 0.592 and 0.487; significance, *p* = 0.002 and 0.021, respectively). Thus, the activity of serum ALT and LDH together with lactate concentrations seems to be a sensitive indicator of the fish responses to DON. The symptoms of carp exposed to DON resemble the situation of freshwater snakehead fish, *Channa punctatus,* that were treated with sublethal concentrations of a carbamate pesticide, leading to increases of LDH and decreased SDH activity in several organs and hyperglycaemia and hyperlactaemia, which suggested that anaerobic metabolism was favored [57].

## 3. Experimental Section

### 3.1. Preparation of Feeds and Husbandry

Ingredients were chosen so that no cereals were included in the experimental diets to exclude cereal-based *Fusarium* toxin contamination of these diets. For the preparation of experimental feeds, all ingredients listed in Table 1 were mixed thoroughly [17]. The feeds were artificially contaminated by adding deoxynivalenol (DON, dissolved in ethanol; purity >98%, lot no. 011M4065V) to the fish oil during the feed preparation process, achieving different concentrations (low dose, 352 μg kg^−1^, medium dose, 619 μg kg^−1^, and high dose, 954 μg kg^−1^, final feed, respectively) [17]. The preparation of the different diets was repeated three times in a pelletizer (L 14-175, Amandus Kahl, Reinbek, Germany) to allow the homogenous distribution of ingredients. The manufactured 4-mm pellets were allowed to cool down to room temperature for two hours before storage at 4 °C until use. The composition of the diets was analyzed by using standard methods. Experimental diets were analyzed for dry matter (DM) (105 °C, until constant weight), crude ash (550 °C, 2 h.), crude fat (Soxtec HT6, Tecator, Höganäs, Sweden) and crude protein content (N × 6.25; Kjeltec Auto System, Tecator, Höganäs, Sweden). Nitrogen-free extract and fibres (NFE) are summarized as shown in Equation (2).

Nitrogen free extract + fibre, (NFE) = 100 − (% protein + % fat + % ash)
(2)

Carp were raised from eggs in our facilities and used for the experiments at 12–16 cm in total length. The fish were kept at a 16 h light/8 h dark photoperiod at 25 ± 0.2 °C (mean ± SD) and acclimatized to the tanks that were integrated into a flow-through system providing 6 L fresh and conditioned water per hour per tank prior to the experiments for three weeks.

### 3.2. Chemicals

All chemicals were obtained from Sigma-Aldrich (Buchs, Switzerland), unless indicated otherwise.

### 3.3. Experimental Feeding Design

The prepared pellets (4 mm in diameter) were given at 2% of body mass once every day to juvenile carp, which were separated into four different feeding groups (control, low dose, medium dose and high dose) with 6 fish each in quadruplicate 54-L tanks for each treatment. The maintenance of optimal rearing conditions (dissolved oxygen, water temperature, pH, conductivity) was controlled during the entire experiments [17]. All experimental procedures have been approved by the cantonal veterinarian authorities of Basel-Stadt (Basel, Switzerland) under permission number 2410. Feed amounts per tank were adjusted to the increased weight on a weekly basis. Uptake of feed was observed in all groups within less than 30 min after offering the experimental diets. Fish were fed the experimental diets for four weeks, and one half of the fish were sampled by using all fish from two tanks of each treatment group. All remaining fish were fed the uncontaminated control diet for a further two weeks before termination of the experiment, in order to investigate the possible reversal of the DON effects. Sampling of fish included blood sampling, recording of weight and length, as well as sampling of individual organs. The calculation of condition factors was achieved according to Equation (3).

Condition factor = weight/(length)^3^ (3)

### 3.4. Histological Determination of Glycogen and Histopathological Scoring

Histological assessments were conducted on liver (*n* = 6 fish per treatment level per sampling day). Tissues were automatically processed (TP1020 tissue processor, Leica Microsystems AG, Switzerland), and at least six sections per fish (3 μm-thick) were mounted on microscope slides and stained with haematoxylin and eosin (HE). Additionally, sections were stained with PAS or Prussian blue to analyze glycogen content and to detect changes in tissue iron content related to erythrocyte turnover. For histopathological examination (Nikon Eclipse 400 microscope), sections were examined in detail at 400× magnification. Quantitative analyses, as described below, were conducted on digital images, which were taken with a Nikon DXM 1200 F digital camera and Nikons ACT-1 software V2.63. Damage to liver tissue was estimated by histopathological scoring in 10 HE-stained sections per fish using semi-quantitative assessments of the severity (0 = no alterations, 1 = mild alterations, 2 = moderate alterations, 3 = severe alterations) according to the suggestions of Zodrow *et al.* [58]. Glycogen content of 5 PAS-stained sections per fish was analyzed by determining the luminosity and RGB_max_. Luminosity can be deduced from the histogram settings in Adobe Photoshop. RGB_max_ was achieved by lightening the green and blue channels in the RGB space. Chrominance was calculated by subtracting the minimal RGB value (which was obtained by darkening the minimal tones) from the RGB_max_ (Adobe^®^ Photoshop^®^ CS3 Extended version 10.1).

### 3.5. Lipid Peroxidation Assay and LDH Activity Measurements

Tissue samples were homogenated in 19 volumes PBS containing 0.1% (w/v) butylated hydroxytoluene (BHT), homogenized using an UltraThurrax (IKA Werke, Staufen, Germany) for 10 s and centrifuged for 10 min at 10,000× g at 4 °C (Centrifuge 5415R, Eppendorf, Basel, Switzerland). The supernatant was used for the TBARs assay [59] with the following modifications. A volume of 40 μL of supernatant was mixed with 200 μL TBARs solution [60], containing 3.75 mg mL^−1^ thiobarbituric acid (TBA), 20% (w/v) trichloric acid (TCA), 9.1 μL mL^−1^ hydrochloric acid (37%),0.06% (w/v) BHT and 866.9 μL distilled water. Thereafter, samples were incubated at 70° C for 90 min, cooled to room temperature and centrifuged at 16,000× g for 15 min at room temperature. In parallel, standards containing 0 to 3200 nM malondialdehyde (MDA) were prepared. Optical densities of all samples were read at 532 nm (Infinite M200, Tecan Group Ltd., Männedorf, Switzerland). Aliquots of the tissue homogenate were also used for the lactate dehydrogenase (LDH) assay and protein determinations. The latter were conducted using the bicinchoninic acid (BCA) assay (Sigma), according to the manufacturer`s protocol. The activity of LDH in tissue homogenates and serum samples was measured according to Bergmeyer [61]. In short, 164 μL NADH solution (0.244 mmol L^−1^ in Tris-NaCl solution (Tris, 81.3 mmol L^−1^; NaCl, 203.2 mmol L^−1^ pH 7.2)) were mixed with 33 μL pyruvate solution (9.76 mmol L^−1^ in Tris-NaCl solution). The reaction was started by the addition of a 20 μL sample, and an absorption decrease at a wavelength of 339 nm was recorded for 10 min (Infinite M200, Tecan Group Ltd., Männedorf, Switzerland).

### 3.6. Nutrient Allocation in Fish

Fish from each group were killed by an overdose of anaesthetic, cut into small pieces with scissors and blended. Homogenates were then dried at 105 °C for 24 h and dry mass was noted. Fat content in dried homogenates was analysed in duplicate by petroleum ether extraction using a Soxhlet apparatus (Soxtec System HT, Tecator, Sweden).

The nutritional status of snap-frozen liver and muscle samples was analysed using different methods, as follows. To amounts of 100 to 250 mg, 1 mL distilled water was added. Liver samples were homogenated with an UltraThurrax (IKA Werke, Staufen, Germany) for 10 s, and the muscle samples were homogenized manually by using a glass potter (Wheaton™ Potter-Elvehjem Tissue Grinders, purchased from Fisher Scientific, Reinach, Switzerland). For the determination of total lipid content, samples were extracted with chloroform-methanol (2:1) containing 0.01% BHT as an antioxidant, according to the method of Bligh and Dyer [62], followed by analyses of total lipids with the sulfo-phospho-vanillin method [63]. A standard curve was prepared with olive oil dissolved in ethanol.

The energy content of dried homogenates was analysed using an IKA C 200 bomb calorimeter. By this method, the dried sample is wrapped in combustible paper and placed in a sealed iron bomb, where its explosive combustion is unleashed by an electric flash, under an O_2_-saturated atmosphere. The bomb is placed in a water bath, which absorbs the heat generated during the combustion; the increase in the temperature of water relates to the heat generated during the combustion. The device is calibrated with a standard compound, benzoic acid (energy released upon combustion: 26,460 Joules g^−1^). Obtained values (in Joules g^−1^ dry matter) were converted to values relative to the fresh weight of fish (% g fish^−1^).

### 3.7. Measurement of Ascorbate

Ascorbate concentrations were analyzed in medium samples and cell extracts according to the method by Vislisel *et al.* [64]. Tissue samples were homogenized in 19 volumes of PBS, after which methanol and diethylenetriaminepentaacetic acid (DTPA) were added to achieve final concentrations of 60% (v:v) and 250 μM, respectively. Samples were centrifuged at 16,000 rpm for 2 min at 4 °C (Centrifuge %427 R, Eppendorf, Basel, Switzerland). Samples of 40 μL from the supernatant were used for the assay. Ascorbate in the samples was first oxidized to dehydroascorbate by the addition of 40 μL tempol (4-hydroxy-2,2,6,6-tetramethyl-piperidinyloxy, 2.3 mM in 2 M sodium acetate buffer) per well, followed by short shaking and the addition of 25 μL *o*-phenylenediamine (OPDA, 5.5 mM in 2 M sodium acetate buffer). Fluorescence emission values were recorded at 450 nm immediately using a plate reader (Infinite M200, Tecan Group Ltd., Männedorf, Switzerland) with excitation at 345 nm. Standard curves were prepared with ascorbate diluted to 14 different concentrations ranging from 0 to 150 μM using the methanol-water mixture containing DPTA.

### 3.8. Preparation of Serum Samples and Determination of Glucose and Lactate

Serum was immediately prepared from blood samples taken with heparinised syringes. Serum samples were stored at −80°C until analyses. Tissue homogenates were centrifuged at 10,000 × g for 10 min, and the supernatant was used for the analysis of glucose and lactate. Glucose was analysed according to the glucose oxidase method. Therefore, samples were mixed with sodium acetate buffer (2 M, pH 5.5) containing 0.1 mg mL^−1^ o-dianisidin, 4 U mL^−1^ glucose oxidase and 2.54 purpurgallin units of peroxidase (from horseradish) per mL. Plates were incubated at 37 °C for 30 min, and the reaction was stopped by the addition of 100 μL 12 N sulphuric acid to each well. Optical densities were read at 540 nm (Infinite M200, Tecan Group Ltd., Männedorf, Switzerland).

Lactate in serum and tissue samples was determined according to Maughan [65] using hydrazine buffer (1.1 mM, pH = 9.0) and 227 μM NAD, and 125 U LDH from porcine heart per well. After incubation for 30 min at room temperature, optical densities were measured at 339 nm and lactate concentrations were calculated from a standard curve prepared with serial dilutions from a lactate standard (998 ± 6 mg L^−1^).

### 3.9. Measurement of Total Protein and Albumin in Serum

Total protein contents were analysed from a diluted serum sample using the bicinchoninic acid (BCA) assay (Sigma), according to the manufacturer’s protocol. Albumin was determined using the bromocresol green (BCG) method, as described by Doumas *et al.* [66], with the following modifications: BCG was solubilised in 0.1 N NaOH and diluted with distilled water and succinate buffer (0.1 M, pH 4.0) at a ratio of 1:3 (v/v). This BCG working solution was added to 25 mL of serum, followed by incubation for 10 min at room temperature and measurement of optical densities at 628 nm (Infinite M200, Tecan Group Ltd., Männedorf, Switzerland). A standard curve was prepared using essential globulin-free bovine serum albumin.

### 3.10. Measurement of AST, ALT and SDH Activity

Activities of aspartate aminotransferase (AST) and alanine aminotransferase (ALT) from samples of 20 μL of serum or tissue homogenate were determined according to the modified methods described by Casillas *et al.* [67] after incubation for 30 min at room temperature by monitoring NADH oxidation at 339 nm (Infinite M200, Tecan Group Ltd., Männedorf, Switzerland). Sorbitol dehydrogenase (SDH) activity in serum samples was measured as described by Bergmeyer [68] using fructose as the substrate. Absorption changes at a wavelength of 339 nm were recorded for 10 min (Infinite M200, Tecan Group Ltd., Männedorf, Switzerland).

### 3.11. Statistics

Data are presented as the mean ± standard error of the mean (SEM), unless indicated otherwise. Coefficients of variance were calculated as shown in Equation (4).

Coefficient of variance (*CV*) = standard deviation/mean × 100 (4)

The effects of the treatments were determined by the comparison of treatment groups to controls using non-parametrical Mann-Whitney U-tests (SPSS 9.0 for Windows). Relationships between parameters were evaluated using Spearman correlation tests. A *p*-value of <0.05 was accepted as being statistically significant.

## 4. Conclusions

The histological alterations in carp livers of DON-treated fish suggest that the fish may face a metabolic crisis caused by tissue damage. The results indicate that oxidative stress leading to lipid peroxidation is involved in the detrimental effects of DON feeding. Taken together, the chemical body composition of the experimental fish in the present study was influenced by the abovementioned factors and gives strong evidence for an influence of DON on the nutritional status of carp. Similar metabolic disorders, including impairment of hepatic metabolism of fats due to oxidative stress, have also been observed in pesticide-treated carp [15]. Nevertheless, the specific biological action and molecular action of DON on liver function in fish is still unclear and needs to be elucidated in further studies.
